# Measurement properties of the benign prostatic hyperplasia impact index in tadalafil studies

**DOI:** 10.1186/1477-7525-8-131

**Published:** 2010-11-12

**Authors:** Mallik Angalakuditi, Rita F Seifert, Risa P Hayes, Michael P O'Leary, Lars Viktrup

**Affiliations:** 1 Lilly Research Laboratories, Eli Lilly & Co, Indianapolis, IN, USA; 2 The Lewin Group, Falls Church, VA, USA; 3 Dept of Surgery, Harvard Medical School, Brigham and Women's Hospital, Boston, MA, USA

## Abstract

**Background:**

To assess the measurement properties of the Benign Prostatic Hyperplasia Impact Index (BII) for use in men with Lower Urinary Tract Symptoms (LUTS) secondary to Benign Prostatic Hyperplasia (BPH) treated with tadalafil.

**Methods:**

Data from a dose-titration (Study 1) and a dose-finding placebo-controlled (Study 2) tadalafil studies of men 45 years of age or older with moderate to severe LUTS (N = 281; N = 1053) were included in this post-hoc analysis. Measures included the BII, International Prostate Symptom Score (IPSS), IPSS Quality of Life Index (IPSS-QoL), LUTS Global Assessment Question, uroflowmetry measure peak flow rate (Q_max_) and postvoid residual volume (PVR). Spearman rank and Pearson correlation coefficients were computed between the BII score and the other measures at each visit. Wilcoxin two-sample tests, t-tests and general linear modeling compared BII scores of subjects with global ratings of improvement versus no improvement, and subjects taking tadalafil versus placebo. Effect size, standardized response mean and Guyatt's responsiveness statistic were calculated for BII and IPSS change scores.

**Results:**

There were high correlations between BII and IPSS & IPSS-QoL and low correlations between BII and Q_max _& PVR at each visit. There were significant differences in BII at the End-of-Study Visit between subjects reporting improvement versus subjects reporting no improvement (Studies 1 and 2, *P *< .0001) and subjects taking tadalafil versus subjects taking placebo (Study 1, *P *= .0045; Study 2, *P *= .0064). The BII and IPSS were both responsive to change.

**Conclusions:**

Results show that the BII is reliable, shows responsiveness to change in patients with BPH-LUTS, and demonstrates construct validity.

## Background

Benign prostatic hyperplasia (BPH) is commonly found in aging men and is characterized by the presence of stromal and epithelial cell hyperplasia beginning in the periurethral zone of the prostate [1-4]. BPH becomes a clinical entity when associated with lower urinary tract symptoms (LUTS); the most common manifestation of BPH [1,2]. Patients with BPH-LUTS experience a significant deterioration in quality of life because of their condition, reporting changes in sleep patterns, anxiety and embarrassment, altered mobility, changes in leisure, daily-living and sexual activities and in satisfaction with sexual relationships [3]. In some men, the progressive enlargement of the prostate may lead to worsening symptoms, acute urinary retention, and consequently, surgical intervention [3].

The primary goal for treating men with BPH-LUTS is usually to relieve symptoms and the bother they cause [5]. In patients with moderate to severe bothersome symptoms treatment options include medical therapies, such as alpha (α) blockers, or in men with an enlarged prostate, 5-α-reductase inhibitors as monotherapy or in combination [4-6].

The most widely employed and validated scoring system for quantifying and monitoring of BPH-LUTS is the 7-item American Urological Association (AUA) Symptom Index developed by the Measurement Committee of the AUA [7]. This instrument measures the severity of voiding and storage symptoms (see Appendix 1) and is the first 7 items of the International Prostate Symptom Score, referred to in this article as the IPSS (see Appendix 1).

The AUA committee also developed the BPH Impact Index (BII) to assess the impact of BPH symptoms on patient health and functioning [8]. The BII is a self-administered questionnaire with 4 questions about urinary problems during the past month regarding physical discomfort, worry about health, how bothersome symptoms are, and whether the symptoms are interfering with doing usual activities (see Appendix 2).

The BII has successfully demonstrated responsiveness to change in patients with BPH-LUTS who were being treated with terazosin versus placebo [9] and dutasteride versus placebo [10,11]. The BII has demonstrated the ability to detect significant differences between men with symptomatic benign prostatic obstruction (with and without indwelling catheter) before and after intervention [12]. Changes in BII scores were greater for BPH-LUTS patients who reported overall global improvement compared to those reporting only moderate, slight, or no improvement [13].

New classes of drugs are currently under investigation for the treatment of men with BPH-LUTS, one being tadalafil, a long-acting phosphodiesterase type 5 (PDE-5) inhibitor used for men with erectile dysfunction (ED). Several reports have shown possible links between BPH-LUTS and ED in epidemiologic, pathophysiologic, and treatment aspects [14].

The initial validation of the BII was carried out 15 years ago by Barry et al [8]. Since that time, the nature of study populations, study designs and treatment options have changed. The BII is an evaluative index useful in measuring the magnitude of change in the impact of BPH-LUTS within a person over time. Its usefulness in BPH treatment is dependent upon it being reliable, responsive to change, and valid. Therefore, it is important to evaluate the construct validity of the BII for patients receiving newer medical treatment such as tadalafil.

## Methods

The BII was administered in 2 clinical studies. Study 1 was a proof-of-concept, randomized, double-blind, placebo-controlled, parallel-design 12-week dose-titration study. Study 2 was a randomized, 5-group, double-blind, placebo-controlled, parallel-design dose-finding 12-week study. Men who were at least 45 years of age, with moderate to severe LUTS due to BPH and evidence of bladder obstruction, were eligible to participate in both studies. Details of the study designs and populations have previously been published [15,16]. The two studies were in compliance with the Helsinki Declaration.

In each study, subjects were screened at Visit 1. If necessary, they started a 4-week washout of BPH treatments; otherwise subjects returned in approximately 1 week. At Visit 2, subjects were required to have an IPSS ≥ 13 and an uroflowmetry measure of peak flow rate (Q_max_) ≥ 4 to ≤ 15 mL/second on a voided volume of 125 mL to continue in the study. Each study included a 4 week single-blind, placebo run-in period to assess treatment compliance and establish baseline levels at its conclusion. At Visit 3 (Week 0), baseline measures were obtained and subjects were randomly assigned to treatment.

Treatment in Study 1 was either tadalafil 5 mg for 6 weeks followed by tadalafil 20 mg for 6 weeks, or placebo for 12 weeks. Subjects returned on Visit 4 (Week 6), and Visit 5 (Week 12), which was the End-of-Study Visit.

Treatment in Study 2 was tadalafil 2.5, 5, 10, 20 mg, or placebo in a 1:1:1:1:1 ratio. The treatment period lasted 12 weeks. Subjects returned on Visit 4 (Week 4), Visit 5 (Week 8), and Visit 6 (Week 12), which was the End-of-Study Visit.

For both studies, subjects completed the BII at every visit starting at Visit 2. All questions asked were about problems over the past month. The first 3 questions of the BII were scored 0 to 3, while the fourth was scored 0 to 4. The sum of the questions produced a BII score that ranged from 0 to 13, with a higher score indicating a worse health impact of BPH symptoms (Appendix 2).

Other questionnaires assessing BPH-LUTS included the IPSS, IPSS-Quality of Life (QoL) and the Global Assessment Questionnaire (GAQ). The IPSS is a validated 7-item urinary symptom severity scale about symptoms occurring over the past month. Scores ranged from 0 to 35 with a higher score indicating more severe symptoms. The IPSS-QoL is a single question: "If you were to spend the rest of your life with your urinary condition just the way it is now, how would you feel about that?" with scores of 0 (delighted), 1 (pleased), 2 (mostly satisfied), 3 (mixed about equally satisfied and dissatisfied), 5 (mostly dissatisfied), and 6 (terrible). The GAQ is a global measure of improvement and was asked at the End-of-Study Visit: "Has the treatment you have been taking during this study improved your urinary symptoms?" with patients responding yes or no.

Objective measures included measuring peak urine flow rate (Q_max_), and postvoid residual volume (PVR), which assesses lower urinary tract function. Both measures are often included in clinical trials in men with BPH-LUTS; however, both are considered optional by the AUA following the initial evaluation of patients for BPH-LUTS [4].

### Statistical Analyses

Construct validity is the ability of an instrument to measure the degree to which an individual possesses a hypothetical trait or quality. Construct validity may be measured by an instrument's strong relationship with other instruments that are intended to measure the same concept (convergent validity) and a lesser relationship with other instruments that measure different concepts (discriminant validity).

The data from each study was analyzed separately. For all analyses, unless explicitly mentioned, subjects were included regardless of what type of treatment they received.

Internal consistency reliability was assessed using Cronbach's alpha statistic [17]. Cronbach's alpha > .70 is considered acceptable, > .80 good and > .90 excellent [18]. Spearman rank correlation coefficients and Pearson correlation coefficients were computed between the BII, IPSS, IPSS-QoL, Q_max _and PVR at each visit. Correlations demonstrating validity typically range from .30 to .80 [19]. Expectations were that BPH-LUTS severity (i.e. IPPS) and related bothersomeness (i.e. IPSS-QoL), which measure concepts similar to BII, would be more highly correlated with BII than the objective measures (i.e., Q_max _and PVR), which measure different concepts. To demonstrate known-groups validity and test for differences between groups expected to be different after treatment, Wilcoxin two-sample tests and t-tests were used to compare BII scores at the End-of-Study Visit between subjects with global ratings of improvement versus no improvement on the GAQ, and between subjects taking tadalafil versus subjects taking placebo. SAS 9.1 for Windows General Linear Models (GLM) Procedure was used for comparisons similar to the t-tests, with the addition of initial or pre-treatment BII score as a covariate. The percentage of men who received tadalafil and improved, and the percentage of men who received placebo and improved were computed for each study. Effect size, standardized response mean, and Guyatt's responsiveness statistic were calculated using BII and IPSS change scores (End-of-Study Visit score minus Visit 2 score) to compare the responsiveness, or the ability to detect change, of the 2 measures [20]. Values of .20, .50, and .80 or greater indicate small, moderate, and large responsiveness, respectively [21].

## Results

### Study 1 Subjects

A total of 281 men met the entry criteria and completed the BII at Visit 2. The mean age among those randomized at Visit 3 was 61.5 years (SD = 8.8, range from 45 to 82). Participants were enrolled from 21 study sites across the United States. A total of 55.2% had BPH-LUTS for more than 3 years. After the placebo run-in period, 278 completed the BII at randomization Visit 3. At Visits 4 and 5, 270 and 259 men, respectively, completed the BII.

### Study 2 Subjects

A total of 1053 men met the criteria and completed the BII at Visit 2. The mean age among those randomized at Visit 3 was 62.1 years old (SD = 7.9, range from 45 to 92). Participants for Study 2 were enrolled from 92 study sites across 10 countries. A total of 51.4% had BPH-LUTS for more than 3 years. After the placebo run-in period there were 1052 who completed the BII at Visit 3 at randomization. Visits 4 through 6 included 1016, 937, and 896 men, respectively, who completed the BII.

### Reliability

Cronbach's alpha for BII was computed at each visit and ranged from .78 to .85 for Study 1 and .81 to .86 for Study 2. Test-retest reliability was not evaluated due to potential deterioration of the patient condition during the washout period. However, the first and second time BII was administered occurred before and after the 4-week placebo run-in for all subjects. Spearman rank correlation coefficients between BII Visit 2 and BII Visit 3 were *r_s _*= .63 for Study 1 and *r_s _*= .64 for Study 2; Pearson correlation coefficients were *r*= .65 and *r*= .65 respectively.

### Validity

For both studies, the initial BII, IPSS, IPSS-QoL, Q_max _and PVR assessment used in the analyses were at Visit 2. Table 1 displays the Spearman rank correlation coefficients between the BPH measures at each visit by study. The BII and IPSS-QoL are all subjective measures of symptoms and/or impact of BPH-LUTS. The IPSS is a urinary symptom severity scale that measures the severity of symptoms of BPH_LUTS. At each visit of Studies 1 and 2, the BII correlated well with IPSS (*r_s _*= .39 to .67) and IPSS-QoL (*r_s _*= .56 to .70). The other 2 variables were objective measures of BPH-LUTS (Q_max _and PVR). At each visit of Studies 1 and 2, BII had very low correlations with Q_max _(*r_s _*= -.13 to .01) and PVR (*r_s_*= .00 to .14). Pearson correlations were very similar.

**Table 1:** Spearman Rank Correlation coefficients among BPH Measures at each visit

Study 1						Study 2					
**Visit 2**		**BII**	**IPSS**	**IPSS-QoL**	**Qmax**	**Visit 2**		**BII**	**IPSS**	**IPSS-QoL**	**Qmax**

	**IPSS**	0.39					**IPSS**	0.51			

	**IPSS-QoL**	0.63	0.42				**IPSS-QoL**	0.56	0.55		

	**Qmax**	0.01	-0.12	0.05			**Qmax**	-0.01	-0.10	-0.02	

	**PVR**	0.00	0.11	0.06	-0.12		**PVR**	0.03	0.16	0.06	-0.21

**Visit 3**		**BII**	**IPSS**	**IPSS-QoL**	**Qmax**	**Visit 3**		**BII**	**IPSS**	**IPSS-QoL**	**Qmax**

	**IPSS**	0.46					**IPSS**	0.53			

	**IPSS-QoL**	0.61	0.53				**IPSS-QoL**	0.56	0.56		

	**Qmax**	-0.01	-0.16	-0.01			**Qmax**	-0.05	-0.16	-0.10	

	**PVR**	0.03	0.09	0.02	-0.19		**PVR**	0.05	0.13	0.11	-0.16

**Visit 4**		**BII**	**IPSS**	**IPSS-QoL**	**Qmax**	**Visit 4**		**BII**	**IPSS**	**IPSS-QoL**	**Qmax**

	**IPSS**	0.57					**IPSS**	0.60			

	**IPSS-QoL**	0.65	0.59				**IPSS-QoL**	0.62	0.66		

	**Qmax**	-0.08	-0.24	-0.13			**Qmax**	-0.12	-0.24	-0.17	

	**PVR**	0.01	0.05	0.04	-0.10		**PVR**	0.12	0.18	0.12	-0.18

**Visit 5**		**BII**	**IPSS**	**IPSS-QoL**	**Qmax**	**Visit 5**		**BII**	**IPSS**	**IPSS-QoL**	**Qmax**

	**IPSS**	0.64					**IPSS**	0.66			

	**IPSS-QoL**	0.66	0.64				**IPSS-QoL**	0.67	0.70		

	**Qmax**	-0.08	-0.17	-0.14			**Qmax**	-0.09	-0.28	-0.18	

	**PVR**	0.00	0.08	0.01	-0.02		**PVR**	0.11	0.17	0.14	-0.23

						**Visit 6**		**BII**	**IPSS**	**IPSS-QoL**	**Qmax**
						
							**IPSS**	0.67			
						
							**IPSS-QoL**	0.70	0.73		
						
							**Qmax**	-0.13	-0.25	-0.23	
						
							**PVR**	0.14	0.21	0.20	-0.22

To demonstrate known-groups validity for BII in each study, subjects who indicated on the GAQ that treatment had improved their symptoms at the End-of-Study Visit were compared to subjects who indicated no improvement. Table 2 displays the 25^th ^percentiles, medians, 75^th ^percentiles, means and standard deviations for improved and not improved subjects. Baseline scores are shown also. There was a significant difference between improved and not improved subjects for both studies (Study 1: Wilcoxin two-sample test *z *= 5.03, *P *< .0001, t-test (*df = *256) = 5.16, *P *< .0001; Study 2: Wilcoxin two-sample test *z *= 10.24, *P *< .0001, t-test (*df *= 469) = 10.64, *P *< .0001). The GLM results were very similar to the t-tests (Study 1 least-squares means: Improved = 2.91, Not improved = 4.97; Study 2 least-squares means: Improved = 2.90, Not improved = 5.04).

**Table 2:** BII Scores for subjects with GAQ Global Ratings of "Improved" versus "Not improved" at End-of-study Visit

Study 1						
		**25th Percentile**	**Median**	**75th Percentile**	**Mean**	**SD**

**Improved**	**Baseline**	5.00	7.00	8.00	6.50	2.81

**(n = 121)**	**End-of-Study**	1.00	3.00	5.00	3.08	2.64

**Not improved**	**Baseline**	4.00	6.00	8.00	5.91	2.80

**(n = 137)**	**End-of-Study**	3.00	5.00	6.00	4.81	2.72

**Study 2**						

		**25^th ^Percentile**	**Median**	**75th Percentile**	**Mean**	**SD**

**Improved**	**Baseline**	4.00	6.00	8.00	5.74	2.98

**(n = 608)**	**End-of-Study**	1.00	2.00	4.00	2.87	2.55

**Not improved**	**Baseline**	4.00	6.00	8.00	5.94	3.00

**(n = 283)**	**End-of-Study**	3.00	5.00	7.00	5.11	3.08

To demonstrate differences between groups that are expected to be different after treatment, BII scores at the End-of-Study Visit for subjects taking tadalafil were compared with subjects taking placebo in each study. Table 3 displays the 25^th ^percentiles, medians, 75^th ^percentiles, means and standard deviations for tadalafil and placebo subjects. Baseline scores are shown also. There were significant differences between tadalafil and placebo subjects for both studies (Study 1: Wilcoxin two-sample test *z *= 2.84, *P = *.0045, t-test (*df *= 254) = 2.65, *P *= .0085; Study 2: Wilcoxin two-sample test *z *= 2.73, *P = *.0064, t-test (*df *= 892) = 2.68, *P *= .0076). Since tadalafil treatment started at Visit 3, GLM was used to compare the 2 groups while controlling for BII at Visit 3. Results were very similar to the t-tests (Study 1 least-squares means: tadalafil = 3.61, placebo = 4.39; Study 2 least-squares means: tadalafil = 3.46, placebo = 4.03).

**Table 3:** BII scores at End-of-Study visit for subjects taking Tadalafil or placebo

Study 1						
		**25^th ^Percentile**	**Median**	**75th Percentile**	**Mean**	**SD**

**Tadalafil**	**Baseline**	3.00	5.00	7.00	4.98	2.99

**(n = 125)**	**End-of-Study**	2.00	3.00	5.00	3.54	2.78

**Placebo**	**Baseline**	3.00	5.00	7.00	5.21	2.73

**(n = 131)**	**End-of-Study**	3.00	4.00	6.00	4.46	2.78

**Study 2**						

		**25^th ^Percentile**	**Median**	**75th Percentile**	**Mean**	**SD**

**Tadalafil**	**Baseline**	3.00	5.00	7.00	4.73	2.88

**(n = 707)**	**End-of-Study**	1.00	3.00	5.00	3.45	2.88

**Placebo**	**Baseline**	3.00	4.00	7.00	4.86	2.95

**(n = 187)**	**End-of-Study**	2.00	4.00	6.00	4.09	2.97

### Responsiveness

Responsiveness statistics were calculated for the BII and the IPSS. The effect size, standardized response mean, and Guyatt's responsiveness statistic for BII were: .78, .79, and 1.02 (respectively) for Study 1 and .74, .75, and .82 (respectively) for Study 2. The corresponding response statistics for the IPSS were: 1.35, 1.15, and 1.31 for Study 1 and 1.39, 1.15, and 1.38 for Study 2. Although the IPSS values were greater than the BII, both measures appeared to be responsive to change.

## Discussion

The BII is a BPH-specific measure that assesses the impact of BPH symptoms on patient health and functioning. Cronbach's alpha for BII ranged from .78 to .85 for Study 1 and .81 to .86 for Study 2, indicating high internal consistency. BII correlated well at each visit with IPSS and IPSS-QoL, measures of BPH symptoms and overall bother of BPH, respectively, which measure concepts similar to BII. These correlations support BII has convergent validity. In contrast, objective measures of BPH such as Q_max _and PVR, which measure different concepts than BII, had very low correlations with BII at each visit. These low correlations offer evidence to support discriminant validity for the BII. It has been recognized for some time in the urological community that urodynamic measures such as Q_max _and PVR are not associated with the symptoms of BPH-LUTS itself [22].

Construct validity also may be established by comparing groups that are known to differ on the concept of interest (known-groups validity) or comparing groups that are expected to differ after experimental manipulation. BII differentiated between subjects who indicated treatment had improved their urinary symptoms compared to subjects with no improvement, lending support for known-groups validity. BII also differentiated between subjects taking tadalafil compared to subjects taking placebo, establishing that BII can distinguish groups that are expected to differ after therapeutic intervention. More subjects receiving tadalafil improved than subjects receiving placebo in both studies. The purpose of the analyses in this manuscript was not to identify who improved on placebo or different doses of tadalafil (2.5 mg, 5 mg, 10 mg or 20 mg), but to conduct a construct validity of a BPH bother score tool. The 2 studies are very different with different dosing and duration of a specific dosing, which makes the studies suitable for validity assessment, but very heterogeneous and therefore unsuitable for comparison.

Our results support 2 validation studies that found strong correlations between BII and IPSS [7,8] and other studies that found strong correlations between BII and IPSS-QoL [11,23,24]. Our findings that BII can differentiate known-groups is consistent with BPH treatment studies that showed BII differentiated between levels of global patient improvement [13], between drug therapy and placebo [10], and between treatment options [11].

The responsiveness statistics calculated for BII indicate that BII is responsive to change. BII should be able to detect when clinically relevant change occurs in a clinical trial or a treatment setting.

The IPSS was the primary efficacy measure in the 2 clinical trials. Given that the BII correlates well with the IPSS, one might question what additional benefit it offers. The correlations between BII and IPSS in Study 1 at Visit 5 (*r_s _*= .64, *r *= .67) and in Study 2 at Visit 6 (*r_s _*= .67, *r *= .68) were strong but they indicated that the IPSS accounted for only 45% and 46% of BII variance, respectively. The BII is a subjective measure and IPSS is a urinary symptom severity measure of BPH-specific health status. While the IPSS addresses specific symptoms that the patient may experience, the BII addresses how BPH symptoms impact the patient. The IPSS-QoL consists of a general question about the impact of BPH on the patient's quality of life. It also had strong correlations with BII in Study 1 at Visit 5 (*r _s _*= .66, *r *= .69) and in Study 2 at Visit 6 (*r _s _*= .70, *r *= .71) but the IPSS-QoL accounted for only 48% and 50%, respectively, of BII variance. The BII measures more specifically the impact of BPH symptoms, including physical discomfort, worry about health, how bothersome symptoms are, and whether the symptoms are interfering with doing usual activities.

When the primary goal for treating patients with clinical manifestations of BPH is to relieve bothersome symptoms, the BII could be used to measure symptom impact on patients. Future research will be necessary to determine if the BII is useful in predicting patient outcomes. This study demonstrates that BII is useful for clinical trials that evaluate drug therapy designed to affect the impact of BPH symptoms on patient health and functioning.

## Conclusions

The results demonstrate that BII is reliable, shows responsiveness, and has construct validity. The BII is a valid instrument to assess the impact of BPH symptoms on health and functioning in clinical trial settings.

## Competing interests

At the time of this study, MA, RH, and LV were employed by Eli Lilly and Company. RFS was employed by The Lewin Group who was contracted by Eli Lilly and Company to conduct this study.

## Authors' contributions

MA was responsible for conception and study design, data interpretation, manuscript writing and overall project management; RFS was responsible for study design, data analysis and manuscript writing; RH, MPO and LV were responsible for study design, data interpretation and critical review of manuscript. All authors read and approved the final manuscript.

## Appendices

## Supplementary Material

Additional file 1 **International Prostate Symptom Score (IPSS)**. Click here for file

Additional file 2 **BPH Impact Index (BII)**. Click here for file
