# Supplementary material for: Measurement properties of the benign prostatic hyperplasia impact index in tadalafil studies
Source: Health Qual Life Outcomes. 2010 Nov 12;8:131. doi: 10.1186/1477-7525-8-131 (PMC2998470; doi:10.1186/1477-7525-8-131)
Supplement: Additional file 1 — International Prostate Symptom Score (IPSS). [file 1477-7525-8-131-S1.PDF]

## Appendix 1. International Prostate Symptom Score (IPSS)

| **In the past month** | **Not at All** | **Less than 1 in 5 Times** | **Less than Half the Time** | **About Half the Time** | **More than Half the Time** | **Almost Always** | **Your Score** |
|---|---|---|---|---|---|---|---|
| **1.Incomplete Emptying**<br>How often have you had the sensation of not emptying your bladder? | 0 | 1 | 2 | 3 | 4 | 5 | |
| **2.Frequency**<br>How often have you had to urinate less than every two hours? | 0 | 1 | 2 | 3 | 4 | 5 | |
| **3.Intermittency**<br>How often have you found you stopped and started again several times when you urinated? | 0 | 1 | 2 | 3 | 4 | 5 | |
| **4.Urgency**<br>How often have you found it difficult to postpone urination? | 0 | 1 | 2 | 3 | 4 | 5 | |
| **5.Weak Stream**<br>How often have you had a weak urinary stream? | 0 | 1 | 2 | 3 | 4 | 5 | |
| **6.Straining**<br>How often have you had to strain to start urination? | 0 | 1 | 2 | 3 | 4 | 5 | |
| | **None** | **1 Time** | **2 Times** | **3 Times** | **4 Times** | **5Times** | |
| **7. Nocturia**<br>How many times did you typically get up at night to urinate? | 0 | 1 | 2 | 3 | 4 | 5 | |

| **Quality of Life due to Urinary Symptoms** | **Delighted** | **Pleased** | **Mostly Satisfied** | **Mixed** | **Mostly Dissatisfied** | **Unhappy** | **Terrible** |
|---|---|---|---|---|---|---|---|
| If you were to spend the rest of your life with your urinary condition just the way it is now, how would you feel about that? | 0 | 1 | 2 | 3 | 4 | 5 | 6 |
