# Supplementary material for: Measurement properties of the benign prostatic hyperplasia impact index in tadalafil studies
Source: Health Qual Life Outcomes. 2010 Nov 12;8:131. doi: 10.1186/1477-7525-8-131 (PMC2998470; doi:10.1186/1477-7525-8-131)
Supplement: Additional file 2 — BPH Impact Index (BII). [file 1477-7525-8-131-S2.PDF]

## Appendix 2.  BPH Impact Index (BII)

| | | | | | |
|---|---|---|---|---|---|
| | **None** | **Only a little** | **Same** | **A lot** | |
| 1. Over the past month, how much physical discomfort did any urinary problems cause you? | 0 | 1 | 2 | 3 | |
| 2. Over the past month, how much did you worry about your health because of any urinary problems? | 0 | 1 | 2 | 3 | |
| | **Not at all bothersome** | **Bothers me a little** | **Bothers me some** | **Bothers me a lot** | |
| 3. Overall, how bothersome has any trouble with urination been during the past month? | 0 | 1 | 2 | 3 | |
| | **None of the time** | **A little of the time** | **Some of the time** | **Most of the time** | **All of the time** |
| 4. Over the past month, how much of the time has any urinary problem kept you from doing the kinds of things you would usually do? | 0 | 1 | 2 | 3 | 4 |
